# Porcine and Chicken Intestinal Epithelial Cell Models for Screening Phytogenic Feed Additives—Chances and Limitations in Use as Alternatives to Feeding Trials

**DOI:** 10.3390/microorganisms10030629

**Published:** 2022-03-16

**Authors:** Hannah Marks, Łukasz Grześkowiak, Beatriz Martinez-Vallespin, Heiko Dietz, Jürgen Zentek

**Affiliations:** 1 Institute of Animal Nutrition, Department of Veterinary Medicine, Freie Universität Berlin, Königin-Luise-Str. 49, 14195 Berlin, Germany; lukasz.grzeskowiak@fu-berlin.de (Ł.G.); beatriz.martinezvallespin@fu-berlin.de (B.M.-V.); zentek.juergen@vetmed.fu-berlin.de (J.Z.); 2 Kaesler Research Institute, Kaesler Nutrition GmbH, Fischkai 1, 27572 Bremerhaven, Germany; heiko.dietz@kaesler.de

**Keywords:** IPEC-J2, epithelial cell line, phytogenic feed additives, in vitro

## Abstract

Numerous bioactive plant additives have shown various positive effects in pigs and chickens. The demand for feed additives of natural origin has increased rapidly in recent years to support the health of farm animals and thus minimize the need for antibiotics and other drugs. Although only in vivo experiments can fully represent their effect on the organism, the establishment of reliable in vitro methods is becoming increasingly important in the goal of reducing the use of animals in experiments. The use of cell models requires strict control of the experimental conditions so that reliability and reproducibility can be achieved. In particular, the intestinal porcine epithelial cell line IPEC-J2 represents a promising model for the development of new additives. It offers the possibility to investigate antioxidative, antimicrobial, anti- or pro-proliferative and antiviral effects. However, the use of IPEC-J2 is limited due to its purely epithelial origin and some differences in its morphology and functionality compared to the in vivo situation. With regard to chickens, the development of a reliable intestinal epithelial cell model has attracted the attention of researchers in recent years. Although a promising model was presented lately, further studies are needed to enable the standardized use of a chicken cell line for testing phytogenic feed additives. Finally, co-cultivation of the currently available cell lines with other cell lines and the development of organoids will open up further application possibilities. Special emphasis was given to the IPEC-J2 cell model. Therefore, all publications that investigated plant derived compounds in this cell line were considered. The section on chicken cell lines is based on publications describing the development of chicken intestinal epithelial cell models.

## 1. Introduction

In view of the increase of antibiotic resistance in animal husbandry, the ongoing restriction of antimicrobials and growing public skepticism about the use of antibiotics and other medications, interest in alternatives of plant origin is steadily increasing [1]. Phytogenic feed additives (PFAs) are defined as substances extracted from plants by technical processes (e.g., steam distillation or cold pressing) and integrated into the diets of livestock in order to improve their performance and health, among other benefits [2]. Whether antimicrobial, antioxidant or performance-enhancing effects, numerous in vitro studies have demonstrated a positive impact of various bioactive herbal additives in pigs and chickens, which is why a wide range of different PFA are already used in animal nutrition today [3,4,5,6,7]. Evidence of efficacy is primarily provided by in vivo feeding trials, but there is a growing interest in the use of different in vitro methods in the development of PFA. Although in vitro methods, unlike animal studies, cannot map the complete metabolism, they allow the determination of several defined parameters such as barrier function [8] or antioxidant potential [9] and are useful to study host–pathogen interactions [10] much more efficiently, cost-effectively and in line with 6R principles [11]. The range of available in vitro models is large and constantly expanding. In addition to other methods such as chemical assays to analyze antioxidant potential [9] or microbial assays such as the broth microdilution method to determine inhibitory potential on microorganisms [12,13,14], the use of different cell lines enables comprehensive studies to investigate new potential feed additives. Since intestinal epithelial cells are in direct contact with the digesta, displaying their interaction with PFA is of great interest. Cultivated epithelial cell lines such as the IPEC-J2 cell line are important in vitro tools for this purpose. This review summarizes the knowledge of the currently available porcine and chicken intestinal cell lines that can be used for screening PFA as well as the methods of assessment that have been established so far. In addition, this work provides an overview of the PFA that have been investigated using these cell models and the observed effects. In particular, the transferability of the results to the in vivo situation is discussed and how this can be optimized in the future. 

## 2. Intestinal Porcine Epithelial Cell Lines

Several different established intestinal porcine epithelial cell lines have already been used to investigate the influence of PFA on the epithelium of pigs. IPEC-1 cells were isolated from the ileum and jejunum and IPEC-J2 cells from the mid-jejunum of an unsuckled piglet less than 12 h old [15]. Both cell lines, as with many other common cell lines such as the human Caco-2 cell line, are immortalized, but non-cancerous and non-transformed, being morphologically and functionally closer to the epithelium of living pigs. In contrast, transformed cell lines are considered to be less vulnerable to stress and cytotoxicity [16]. Beside the lacking ability to form a polarized monolayer, that probably contributes to the fact, that the intestinal porcine epithelial cell line IPI-2I which was isolated from the ileum of an adult boar [17] have not been used for studying PFA so far. The cell line ZYM-SIEC02, similarly to IPEC-1 and IPEC-J2 cells, was isolated from the small intestine of neonatal unsuckled piglets [18], but its use is still limited to a small number of studies. This also applies to the cell line PSI cl 1 which was derived from the small intestine of an adult pig [19]. Thus, these cell lines, as well as the IPI-2I cell line, will not be discussed further in this review.

When cultured, IPEC-J2 and IPEC-1 form a polarized monolayer with tight junctions (TJ), and microvilli located on their apical side [20] and moreover develop extensive metabolic functions. Those features allow measurements of barrier function and nutrient transport [21] as well as investigations on the impact of external challenges as mycotoxins [22], microbial pathogens [10], and their toxins [23,24], heavy metals such as zinc [25] and copper [26]. Gene expression analysis of both cell lines revealed that IPEC-J2 is higher differentiated in morphology e.g., developing a two-fold higher number of microvilli and villin, as compared to IPEC-1, which indicates a more efficient crosslinking between microvilli [21]. Furthermore, whereas IPEC-1 cells seem to be in a non-proliferative state, IPEC-J2 cells show an active aerobic and anaerobic glucose-consumption characterized by a much higher rate of oxidative phosphorylation, intracellular ATP-content, O2- and glucose-consumption as well as lactate production and are therefore more suitable for metabolism-focused investigations [21]. 

### 2.1. Methods to Study Barrier Function in IPEC

The barrier function is one of the most important basic properties of the intestinal epithelium due to its direct contact to the intestinal lumen and the digesta contained therein. Damage to the epithelium integrity alters its permeability, which in normal conditions ensures selective passage of essential substances such as nutrients. As a consequence, microorganisms or their toxins could cross the epithelium and cause local or systemic damage to the host. Some exhaustive reviews about the influence of food components and PFA on intestinal barrier function in animal experiments but also ex vivo and in vitro have recently been published [8,27]. Various methods are available to determine the integrity of the barrier function. One important measurement is the cell viability, i.e., the intactness of the enterocytes, which is commonly measured by 3-[4, 5-dimethylthiazole-2-yl]-2, 5-diphenyltetrazolium bromide (MTT) reduction assay, neutral red method or lactate dehydrogenase release. Moreover, propidium iodide is often used for cell viability assessment, not only for epithelial cells but also for bacteria viability [24,28]. In addition, the electrical measurement of the transepithelial electrical resistance (TEER), which is largely determined by the integrity of the TJ, as well as the paracellular permeability, usually analyzed using fluorescein isothiocyanatedextran are significant indicators of barrier function intactness [16]. The measurements of TEER and epithelial permeability are also influenced by the culture medium. Specifically, the addition of fetal bovine serum (FBS) leads to a lower resistance and higher epithelial permeability in comparison to the use of porcine serum (PS). While a higher TEER induced by FBS is more suitable for investigating the impact of substances expecting to impair barrier integrity, the lower TEER is more comparable to the in vivo situation [16]. Furthermore, TJ ultrastructure as well as the morphology of enterocytes better mimic the normal physiological porcine epithelium when cultured in PS. Polarization markers glucose transporter 2 (GLUT2) and sodium–potassium adenosine triphosphatase (Na+/K+-ATPase) in basolateral membrane are expressed in cells cultured in either PS or FBS but apical membrane polarization markers ezrin and sodium/glucose cotransporter (SGLT) 2 could not be found in FBS-cultured IPEC-J2 cells, probably because of their low cell height [29]. Despite these supposed advantages of PS, the addition of FBS to the medium is far more common, which might be due to the original protocol as well as the lower cost of purchase.

### 2.2. Cellular Transport and Permeability

As part of the regulation of the barrier function of intestinal epithelium, the impact of PFA on transport mechanisms forms another field of interest. In general, the epithelial cell barrier can be passed by compounds through two main routes: The paracellular pathway between cells or the transcellular pathway throughout the cell [30]. Due to the expression of various transport proteins and the formation of tight junctions, as well as the measurability of paracellular permeability, studies of both para- and transcellular transport are also possible in an IPEC-J2 cell model [16]. 

In IPEC-J2 cells challenged with enterotoxigenic *Escherichia coli* (ETEC K88), for example, expression of Na+/H+ exchanger 3 (NHE3) as well as water channel aquaporins were decreased [31], which eventually could be counteracted by PFA which are already known for their ability to reduce ETEC K88 adhesion. The Ussing chamber is a common ex vivo method for determining absorption/transport processes of the epithelium. In most of the protocols, intestinal explants are mounted into the chambers, but in the course of reducing and replacing animal experiments, animal-free alternatives are also being sought here. Mounting IPEC-J2 monolayers into the Ussing chamber demonstrated a positive influence of epidermal growth factor on TEER and the absorption of glucose and glutamine in lipopolysaccharide (LPS)-challenged cells [32], which is why combining IPEC-J2 cells with the Ussing chamber may also offer further information on the influence of PFA on transport processes.

### 2.3. Pathogen Infections

With regard to host–microbial interactions, many similarities between the IPEC-J2 cell line and the porcine epithelium in vivo have been detected. Invasion with the bacterium *Salmonella enterica* ser. Typhimurium in IPEC-J2 cells is comparable to that in porcine ileal mucosal explants [33] and secretion of interleukin (IL) -8 and macrophage inflammatory protein (MIP) 3 alpha increases in both IPEC-J2 cells and orally infected pigs [34]. IPEC-J2 therefore seem to be a suitable cell line for studying antimicrobial effects in vitro. In studies using IPEC-J2 cells and porcine mucosal explants interactions with *E. coli* also showed a high correlation; however, some enterohemorrhagic *E. coli* (EHEC) mutants differed in their adherence [35]. Moreover, there is disagreement as to whether IPEC-J2 cells support the adhesion of the pathogenicity factor F18 of ETEC through the expression of a fimbriae-specific receptor. Some authors stated that IPEC-J2 cells do not express this receptor [36,37], but the expression of the F18 receptor has been the subject of several recent studies [38,39], suggesting that IPEC-J2 cells do express the F18 receptor. It should be furthermore noted that the IPEC-J2 cell line has been isolated from the small intestine, so the influence of specific pathogens or its antigens located in the colon may not be adequately represented. Nevertheless, receptors for some specific toxins physiologically acting in colon are also present in IPEC-J2 cells, as a reaction of IPEC-J2 cells to toxins from *Clostridioides difficile* was recently reported [24]. Certain mechanisms affecting colonic cells in vivo therefore can also be studied in IPEC-2 cells. At present, moreover, no non-cancerous colon cell line (human- or animal-derived) is available [40]. Although co-infections of IPEC-J2 cells, for example with porcine bocavirus and porcine circovirus 2 [41] have been performed, to date, culturing of bacterial community dynamics is not possible, therefore, studying of bacterial–host interactions is limited to single bacterial strains [40]. In vitro digestion models such as the continuous fermentation model PolyFermS give the opportunity to display the interaction of e.g., probiotics and bacterial communities derived from feces [42]. Connecting IPEC-J2 cells to such a digestion model could enable further studies concerning the interplay between host cells and entire heterogeneous bacterial communities and therefore act as an interesting tool for also studying PFA. Due to its similarity in gene expression to the human intestine, the IPEC-J2 cell line offers the opportunity to study interactions with zoonotic pathogens [10,34]. When researching foreign pathogens, on the other hand, it is always important to bear in mind that the response may differ from that in the actual host. For example, Shiga toxin 2e-producing *E. coli* (STEC) strains isolated from humans did not bind to IPEC-J2 cells, unlike isolates from pigs [43], and also rotavirus strains derived from pigs showed a significant higher infectivity in IPEC-J2 cells than those derived from humans [44]. Thus, host specificity plays an important role and should be considered when designing studies on the interactions of epithelial cells with bacteria.

### 2.4. Oxidative Stress

A further field of interest concerning PFA is their anti-oxidative potential, not only for extending the shelf life of feed, but especially for preventing endogenous cells from cell death due to oxidative stress [45]. A favored applicant for experimentally inducing oxidative stress in IPEC-J2 cell line is H_2_O_2_ due to its capability of negatively affecting distribution of TJ proteins e.g., zonula occludens 1 (ZO-1), deteriorating barrier function, decreasing cell viability and triggering intracellular production of reactive oxidative species (ROS), an indicator for oxidative stress [46]. When examining antioxidant properties of PFA, the cell model cannot automatically be transferred to the situation in vivo. Although exogenous oxidative stress can be successfully simulated by adding H_2_O_2_, it should be noted that in addition to exogenous stress, epithelial cells in vivo are exposed to stress of endogenous origin (mainly ROS originating from mitochondrial oxidation), which is not simulated in vitro, nor is the multifactorial oxidation induction by other stimulants such as enterotoxins [47]. The use of the cell model nevertheless offers the possibility of investigating substances in general for their antioxidant activity. 

Most of the studies considered in Section 2.5.5. (“Antioxidative effects”) used H_2_O_2_ for inducing oxidative stress in IPEC-J2 cells. Interestingly, a study from 2016 suggested a xanthine/xanthine oxidase (X/XO)-induced oxidative stress model when investigating apoptosis in weaned piglets. The authors detected, that expression of apoptosis-related genes in (X/XO) challenged cells is more highly correlated to 21-day-old pigs than those in H_2_O_2_-induced models [47].

### 2.5. The IPEC-J2 Cell Line as Model for Investigating PFA 

Due to the above mentioned advantages in function and morphology over the IPEC-1 cell line, IPEC-J2 cells in particular have been used so far for investigations of epithelial reactions to PFA. Cytokine secretion and protein expression of TJ is mainly consistent with those of jejunocytes in vivo [16] and even the inflammatory pathway markers Nuclear factor kappa B (NF-kB) and myeloid differentiation marker 88 (MYD88) are expressed [48]. Nevertheless, the model presents some limitations when comparing with the porcine jejunum. Claudin (CLDN) -2 and -15 are not expressed in IPEC-J2, resulting in a higher ion permeability than in vivo [20,29]. Moreover, although expression of mucin and MUC3 [20] is assumed, it remains unclear if IPEC-J2 cells express MUC2, a major mucus-producing mucin in humans and other animals. Thus, the lack of goblet cells or MUC2 expression have been repeatedly reported [20,49] but very recent studies have detected and measured messenger ribonucleic acid (mRNA) expression of MUC2 [50,51]. Goblet cells could not be detected [20]. Finally, no brush border enzymes have been determined yet, making it impossible to study the effects of pathogen agents or feed materials on those enzymes [16]. For reproducible research with in vitro cell lines, a strictly standardized setting is essential. Differences in seeding density influence the TEER, proliferation capacity and functionality of the cultured IPEC-J2 cells [16]. Therefore, cell density should always be considered in order to reduce variability. The passage number of the culture is a frequently underestimated parameter; it is often not even stated in experiments [16]. When acquiring cell cultures from cell banks, this important information often remains unclear. Although there is little literature on the influence of the passage number on IPEC-J2 cell physiology, it is known that Caco-2 human cell line is clearly affected by the number of passages [52,53]. It has been shown that increasing passage number can lead to a reduced secretion of signaling agents by IPEC-J2 cells, but this might be counteracted by adding PS in the culture media [29]. Thus, in order to obtain reproducible values, the number of passages should always be provided if possible.

Furthermore, it is particularly important to work under strictly sterile conditions in order to prevent contamination of the cells. Important contaminants are *Mycoplasma* spp., which can remain unnoticed for a long time and require complex and cost-intensive disinfection processes when diagnosed [54]. Regular rapid tests for the detection of mycoplasma are therefore advisable as well as examining the identity of cell lines when purchasing from cell banks as it, especially in animal cell lines, occurs to be incorrect [55]. Since PFA also pose a great risk of contamination, they should always be sterile filtered before being tested in cell models. Essential oils in excessively high concentration can have a cytotoxic effect on epithelial cells and the positive effect can thus be transformed into damage to the epithelium. Finding the appropriate concentration of the tested substances is therefore crucial [8]. When it comes to investigations of slightly soluble extracts, dissolving of samples can be a challenging task and although dimethyl sulfoxide (DMSO) or ethanol can be used for bringing them into solution, a concentration above one percent is not recommended [16].

#### 2.5.1. Effects on Barrier Function

Several studies have investigated the impact of PFA on barrier function in IPEC-J2 (Table 1). For instance, peppermint and spearmint oil showed no effect on TEER of IPEC-J2 cells [56]. Resveratrol, a polyphenolic compound abundant in grape skin, was able to counteract decreases in TEER and elevate paracellular permeability. Furthermore, integrity was promoted by an increased expression of CLDN-4 via mitogen-activated protein kinase-dependent (MAPK) pathways in IPEC-J2 cells challenged with mycotoxins deoxynivalenol (DON) [57,58]. As resveratrol has an inhibitory effect on c-Jun-N-terminal kinase (JNK), findings revealed that reduction of endocytosis and degradation of CLDN-4 and TJ associated gene ZO-1 by DON further seems to be regulated by JNK [58]. In a model using a mixture of the mycotoxins DON and fusariotoxin T 2 (T2) to impair barrier function, rosmarinic acid showed the ability to restore TEER and to promote distribution of TJ protein CLDN-1. Levels of pro-inflammatory IL-6 and IL-8 were further decreased, suggesting an anti-inflammatory potential of rosmarinic acid [59]. Another study investigated the influence of several PFA on IPEC-J2 cells after deteriorating barrier function by disrupting TJ via a calcium-switch assay. After the calcium switch, TEER abruptly decreased but, interestingly, cell viability was not affected. After 24 h, all groups of cells showed a recovery in TEER but those pre-treated with liquorice (*Glycyrrhiza glabra*) root extract, angelica (*Angelica archangelica*) root powder and a plant powder mix containing ground gentian (*Gentiana lutea*) root, ground angelica root and ground cinnamon (*Cinnamomum verum*) bark were able to increase the TEER beyond the control group growing in complete medium without any supplementation. Treatment with oak (*Quercus robur*) bark extract, though able to increase TEER 6 h after application, did not improve barrier function compared to control group at a later point of time. Milk thistle (*Silybum marianum*) fruit extract, uncombined ground cinnamon bark and ground gentian root did not affect the TEER. In addition, liquorice root extract increased the expression of the TJ protein CLDN-4 [60].

#### 2.5.2. Effects on Infection with Intestinal Microbial Pathogens

Pathogenic bacteria *Salmonella* Typhimurium and *Salmonella* Choleraesuis are causing immense animal suffering as well as economic losses. Therefore, investigations concerning the impact of PFA on *Salmonella*–epithelium interaction have become a major application of the IPEC-J2 cell line. The impact of PFA on IPEC-J2 cells infected with microbial pathogens are shown in Table 2.

In a study using green fluorescent protein labelled *S.* Typhimurium, a prebiotic derived from yellow pine wood and mainly composed of glucose-galactose-mannose-xylose oligomers showed the ability to reduce *Salmonella* binding to IPEC-J2 cells by 90% per individual cell. In addition, *Salmonella* invasion was decreased after treatment. Whereas pre-exposure to methyl α-d-mannopyranoside also inhibited *Salmonella* invasion, suggesting a contribution of mannose-sensitive adhesions to the mechanism of invasion, methyl α-d-mannopyranoside could not decrease *Salmonella* binding. Binding, therefore, seems to be mediated by other fimbrial adhesions [61]. 

Incubating IPEC-J2 cells with cinnamaldehyde, carvacrol and cinnamic acid also successfully counteracted *Salmonella* invasion. However, bacterial viability as well as motility and development of flagella were not affected. The combination of cinnamaldehyde, cinnamic acid and propionic acid led to structural impairment of adhesive organelle fimbriae, which likely decreased attachment of *S.* Typhimurium. Cinnamaldehyde alone and carvacrol in combination with *S.* Typhimurium and propionic acid furthermore showed an immune stimulating effect by upregulating immune-related gene heat shock protein 70 [62].

Even in sub-lethal doses, carvacrol was not able to influence adhesion of *S.* Typhimurium to IPEC-J2 cells, whereas invasion was successfully reduced due to this concentration. Since expression of antimicrobial peptide porcine beta-defensin (pBD) -2 furthermore was inhibited, the invasion but not adhesion seems to be responsible for its induction [63]. 

In addition to infection with *S.* Typhimurium, *E. coli* poses major challenges to pig production. Numerous studies have already focused on the search for effective antimicrobial plant-based ingredients to combat pathogenic *E. coli*. 

The use of *Lythrum salicaria* herb and its c-glycosylic ellagitannins, castalagin, vescalagin, and salicarinins A and B were tested on their antimicrobial effect and shown to decrease pathogenic *E. coli* adhesion to IPEC-J2 cells. In non-infected cells, TEER and production of TJ proteins CLDN-4 and ZO-1 was increased without impairing cell viability. Interestingly, analysis showed a correlation between TEER and CLDN-4 expression indicating that CLDN-4 is an essential part of monolayer integrity [64].

Peptides derived from soybean meal fermented with *Bacillus subtilis* strain BS12 also increased TEER and expression of several TJ proteins in IPEC-J2 cells challenged with pathogenic *E. coli*. By inhibiting secretion of pro-inflammatory IL-6, IL-8 and IL-1β as well as phosphorylation of NF-κB, inhibitor kappa B-alpha (IκB-α), and p38 MAPK, the peptides moreover showed the ability to reduce gut inflammation [65].

The use of the green tea polyphenol epigallocatechin-3-gallate efficiently suppressed translocation of non-pathogenic *E. coli* across the IPEC-J2 monolayer. The immunological barrier seemed to play an important role in the protective mechanism as levels of pBD-1 and pBD-2 were increased by epigallocatechin-3-gallate, while epithelial physical barrier was not affected. Secretion of pBD-2 was proved to be upregulated via p38 MAPK pathway [66].

An adhesion inhibiting potential has also been demonstrated for locust bean (*Ceratonia silique*) exopolysaccharide and wheat bran [67] whereas soybean hulls, sugar-beet pulp, locust gum, fructooligosaccharides, inulin, mushroom, mannanoligosaccharides, a fermented product from *Aspergillus oryzae* as well as an additive containing thyme extract had no influence on adhesion of pathogenic *E. coli* to IPEC-J2 cells [67,68]. None of the additive was able to protect the IPEC-J2 cells from *E. coli*-induced decrease in TEER [68].

#### 2.5.3. Anti-Inflammatory Effects 

For investigating the ability of PFA to counteract intestinal epithelial inflammation, a pre-stimulation of IPEC-J2 cells using LPS can be performed. LPS are glycolipids located in the outer membrane of gram negative bacteria [69] and are well-known for their inflammation-inducing effect on intestinal epithelium [70]. The effects of PFA on LPS-stimulated IPEC-J2 cells are listed in Table 3.

A blend composed of 75% thymol and 25% cinnamaldehyde (TCB) was investigated concerning its influence on LPS-challenged and unchallenged IPEC-J2 cells. In LPS-challenged cells, increased permeability due to LPS was decreased by TCB and expression of CLDN-4 was promoted, concluding a positive effect of the tested blend on barrier function. Furthermore, secretion of pro-inflammatory cytokines TNF-α and polymeric immunoglobulin receptor (pIgR) decreased while there was an increase in anti-inflammatory cytokine IL-10. The enhancement in epithelial cell integrity, measured by microscope observation of manual inflicted scratches in monolayer regeneration, suggests an acceleration of wound recovery in cells treated with TCB compared to untreated cells. Surprisingly, challenge with LPS also accelerated recovery of epithelium instead of slowing it down. This might be based on the fact that LPS signaling conducts to intestinal reaction to injury [71].

Additionally, not combined with cinnamaldehyde, thymol promoted barrier function of LPS-challenged IPEC-J2 cells and was able to inhibit secretion of pro-inflammatory cytokines (IL-8, TNF-α). Moreover, actin staining displayed a structural stabilization of cytoskeleton integrity and even production of ROS was downregulated. No effect could be determined on expression of nutrient transporters [70].

In LPS-stimulated cells, eugenol, a major component of cloves, successfully decreased pro-inflammatory cytokines IL-8 and tumor necrosis factor alpha (TNF-α) and increased abundance of TJ proteins ZO-1 and occludin (OCLN). Moreover, redistribution of ZO-1 was enhanced and the decrease of numerous nutrient transporters could be counteracted resulting in maintenance of physiological nutrient exchange [72]. Incubating IPEC-J2 cells with garlic (*Allium sativum*) -derived diallyl disulfide (DADS) and diallyl trisulfide (DATS) did not protect epithelial barrier function from decrease in TEER though expression of TJ protein ZO-1 tended to increase. Nevertheless, inhibition in IL-8 secretion suggests an anti-inflammatory influence on LPS-challenged cells [73].

In recent years, several studies have focused not only on the influence of PFA on the inflammatory response to LPS per se, but also on the MAPK and NF-κB signaling pathways that induce inflammation. Astragalus (*Astragalus membranaceus*) polysaccharides decreased gene expression of phosphorylated p38MAPK, extracellular-signal regulated kinases 1/2, NF-κB p65, key proteins of the MAPK/NF-κB inflammatory pathway, while increasing IκB-α, which downregulates the NF-κB pathway [74]. Similarly, alkaloid berberine reduced proteins which are associated with NF-κB/MAPK signaling pathway. This has shown to counteract inflammatory response indicated by dose-dependent suppressed secretion of pro-inflammatory cytokines IL-1β, IL-6 and TNF-α [75].

Three different mycotoxin-induced inflammation models with either DON, T2 or zearalenon (ZEN) were used to investigate the effect of different halophyte extracts (*Convolvulus soldanella*, *Eryngium campestre*, *Galium arenarium*, *Limonium vulgare* and *Ononis spinosa*) on IPEC-J2 cells. While decreased cell viability was counteracted in T2- and ZEN-challenged cells, no protective effect could be determined in IPEC-J2 cells incubated with DON. Furthermore, secretion of pro-inflammatory cytokines TNF-α and IL-8 was elevated in DON- and T2-stimulated cells, which could be counteracted by the halophyte extracts. ZEN had no effect on cytokine production [76].

#### 2.5.4. Antiviral Effects 

Since, in addition to bacterial pathogens, various viral infections also lead to major losses in pig production, the IPEC-J2 cell model has also been increasingly employed in virus research in recent years [41,77,78,79]. Regarding the protective effect of PFA, however, only the alkaloid tomatidine, derived from the skin and leaves of tomatoes, in three different concentrations (1.039; 2.078; 4.157 µg/mL) has been studied. By blocking viral endopeptidase C30 activity, it reduced activity of porcine epidemic diarrhea virus (PEDV) in IPEC-J2 cells [80].

#### 2.5.5. Antioxidative Effects 

Studies on the antioxidant activities of PFA have increasingly attracted attention, especially in recent years. The antioxidative effects of PFA are demonstrated in Table 4. 

Java tea (*Orthosiphon stamineus)* root, stem and leaf extracts showed a beneficial effect on cell viability and could decrease antioxidative stress, indicated by reduced ROS production [81]. Furthermore, a combination of in vitro digested chestnut (*Castanea sativa* Mill.) tannin extract and quebracho (*Schinopsis* spp.) tannin extract (50% each) successfully counteracted antioxidative stress, as demonstrated by increases in cell viability. Moreover, cells pre-treated with dextran sulphide sodium (DSS) for inducing chemical stress were successfully protected from decrease in cell viability by 50–400 µg/mL of applicant. Interestingly, a higher dosage (600–1200 µg/mL) had no beneficial effect on cell viability of DSS-challenged cells [82].

Extracts from Java tea known for its therapeutic effect on kidney diseases in humans lowered secretion of cellular oxidative damage product malondialdehyde (MDA) as well as increasing TEER and expression of several TJ proteins in IPEC-J2 cells [83]. 

Besides a decrease in oxidative damage products, pectic polysaccharides from *Codonopsis pilosula* (CPP-1) and *Codonopsis tangshen* (CTP-1) root, inulin-type fructans from *C. pilosula* (CPPN) and *C. tangshen* (CTPN) radix as well as extracts from *Ulva prolifera* mainly consisting of polyphenols and unsaturated fatty acids and polysaccharides from *Hippophae rhamnoides* (HRP) showed the ability to enhance functionality of at least some antioxidative enzymes such as glutathione peroxidase (GSH-Px), superoxide dismutase (SOD) and chloramphenicol acetyltransferase (CAT) in IPEC-J2 cells [84,85,86,87]. 

Similarly, DADS and DATS also successfully increased activity of CAT in IPEC-J2 cells challenged with H_2_O_2_ [88]. The extracts from *C. pilosula* and *C. tangshen* further increased total antioxidant capacity (T-AOC) as well as the cell viability whereas HRP, in addition to its antioxidant property, was also capable of stimulating the release of various proinflammatory cytokines [85,86,87].

As expression of nuclear factor erythroid 2-related factor 2 (Nrf2) was upregulated in IPEC-J2 cells treated with *U. prolifera* extract or CPP-1/CTP-1, authors conclude that Nrf2 is involved in the upregulation of antioxidant enzymes to a significant extent [84,85]. To examine if Nrf2 signaling due to *U. prolifera* is related to AMP-activated protein kinase (AMPK) pathway, AMPK inhibitor compound C was added to culture. As the antioxidative effects of *U. prolifera* were only partially counteracted and expression of Nrf2 was not affected, dependency of Nrf2 on AMPK pathway could be excluded. For investigating cellular fitness, influence of *U. prolifera* on mitochondrial respiration was determined analyzing oxygen consumption rate (OCR). Results showed *U. prolifera* extract to increase basal and maximal OCR as well as non-mitochondrial respiration and ATP production [84]. 

Moreover, resveratrol counteracted oxidative stress via Nrf2 signaling pathway indicated by increased cell viability, abundance of TJ proteins, activity of antioxidative enzymes SOD, CAT, GSH-Px and decrease in apoptosis and ROS production [89]. Besides promoting cell viability and activity of antioxidative enzymes as well as decreasing secretion of cellular oxidative damage products, alkaloid koumine from *Gersemium sempervirens* counteracted apoptosis due to H_2_O_2_ and reduced the activity of apoptosis-related enzymes caspase-9 and -3, which, amongst others, inhibit pro-apoptotic Bcl proteins. Intrinsically, apoptosis is regulated by the mitochondrial membrane potential, which is controlled by releasing various Bcl proteins. In H_2_O_2_-challenged cells, a reincrease of lowered mitochondrial membrane potential as well as in anti-apoptotic B-cell lymphoma (Bcl) -2 proteins could be determined after treatment with koumine. Moreover the activity of caspase-3 and -9 as well as secretion of ROS and MDA was reduced, while anti-apoptotic enzymes were activated [90]. 

Quercetin similarly showed the ability to protect IPEC-J2 cells from apoptosis due to H_2_O_2_ as reduction of oxidative stress and enhance in barrier function [91] as well as an improvement in cell viability and reversement of changes in apoptosis/necrosis rates was reported [92]. Measurement of lactate dehydrogenase (LDH) activity in culture medium further demonstrated the anti-oxidative effect of quercetin as LDH which physiologically is located inside of enterocytes was released into the medium after damage of cellular membrane [92]. The antioxidative effect of rosmarinic acid was shown in H_2_O_2_-challenged cells [91] as well as in a mycotoxin-induced stress model [59].

Moreover, allicin, odorant component of garlic was not only investigated in H_2_O_2_-challenged IPEC-J2 cells, where it was able to reduce ROS and MDA while increasing the activity of SOD, but also in a so-called endoplasmatic reticulum (ER) stress cell model. By incubating IPEC-J2 cells with tunicamycin, functional homeostasis (e.g., protein folding) of ER was impaired leading to the unfolded protein response (UPR). While apoptosis, inositol-requiring enzyme 1 alpha (IRE-1α), one of the major signaling pathways in UPR, and ER stress signaling protein X-box binding protein 1 (XBP-1) were upregulated by allicin, the second contributing signaling C/EBP homologous protein (CHOP) pathway and cell proliferation could not be affected. As blockage of IRE-1α suppressed the beneficial effects of allicin on tunicamycin-stressed cells such as reduction of ROS and different signaling proteins 78-kDa glucose-regulated protein (GRP78), activating transcription factor 4 (ATF-4), and phosphorylated eukaryotic initiation factor-2 alpha (p-eIF-2α), authors suggest that allicin mainly contributes to intestinal health by activating the IRE-1α signaling pathway [93].

#### 2.5.6. Impact on Cell Proliferation

A further interesting marker in investigations into PFA is cell proliferation, as depending on the objective, a promoting or inhibiting effect on the epithelial cells may be desirable (Table 5). 

To compensate more quickly for the loss of epithelial cells in the course of inflammation on the one hand, increased proliferation can bear a positive effect on the attacked intestine. Polyphenolic quercetin was reported to have an enhancing effect on cell proliferation of IPEC-J2 cells by levelling up proliferation phase S as well as total proliferation index. Interestingly, quercetin showed a negative effect on epithelial regeneration, as analyzed by microscopic examination of manual inflicted monolayer [92]. 

In contrast to the desired pro-proliferative effect to compensate for inflammation-induced cell death, research into new substances for cancer therapy focuses on an anti-proliferative effect on the cells. In this context, extracts of the plant *Periscella frutascens* revealed the ability to reduce the proliferation of IPEC-J2 cells and further decrease the expression of cancer-relevant genes cyclin D1 and 67 kDa laminin receptor, which significantly contributes to the growth and metastasis of tumor cells [95].

### 2.6. The IPEC-1 Cell Line as Model for Investigating PFA

Only few studies have been performed using the IPEC-1 cell line for investigating the impact of PFA on epithelium in vitro. This might be due to lower differentiation in morphology and metabolic functions of IPEC-1 compared to the IPEC-J2 cell line [21], making it less attractive. Nevertheless, a barrier integrity enhancing effect as well as antimicrobial activity could be determined using the IPEC-1 cell line. 

Cinnamaldehyde could improve barrier function characterized by increased TEER, decreased paracellular permeability and promoted localization of TJ proteins CLDN-1 and CLDN-3 to the plasma-membrane. Furthermore, protein expression of neutral and basic amino acid transport protein rBAT (rBAT), cystine/glutamate transporter xCT (xCT) and L-type amino acid transporter 2 (LAT2) were elevated by cinnamaldehyde [96]. In ETEC-challenged IPEC-1 cells, yeast extract, daidzein, bromelain and allicin contributed to barrier integrity by an increase of TEER and decrease of paracellular permeability. Yeast nucleotides, unsaturated oligo-mannuronic acid, ulvan, *Chlorella vulgaris* extract, cinnamaldehyde and carvacrol, on the contrary, could not affect those parameters [97].

## 3. Intestinal Chicken Epithelial Cell Lines

### 3.1. Approaches to Date

In contrast to pigs, no reliable epithelial cell line has been established for chickens to date, although several attempts have been made. Extensive studies on cells isolated from the small intestine of one-day-old chickens revealed an enterocyte-like structure as well as an impact of different chemical stimuli on morphology and viability of the cells. DON, for example, led to cell death and degeneration, as also seen in IPEC-J2 cells, whereas LPS, which leads to a clear reaction in IPEC-J2 cells, had only little influence on the above mentioned chicken cell line. Critically, although ZO-1 could be detected by an immunofluorescence assay, its distribution did not correspond to the expected distribution in vivo. In addition, the cultivation was only carried out for up to six passages [98] but for extensive studies, a self-renewing cell culture with a high number of passage cycles would be necessary.

Similarly, chicken epithelial cell lines from previous establishment approaches did not survive longer than 7–10 days [99,100,101,102,103]. The cells were positive for several epithelial markers (cytokeratin 18, intestinal alkaline phosphatase, intestinal fatty acid-binding protein), but vimentin, characteristically expressed by fibroblasts or endothelial cells could also be detected [104]. It should be noted here that vimentin has been detected in various established epithelial cell lines [105], including IPEC-1 and IPEC-J2 cells [29]. Therefore, even a proof of presence is not a clear exclusion criterion in the development of new cell lines. However, as with IPEC-J2 cells, a dedifferentiation to a mesenchymal cell type should be ruled out for determining the non-carcinogenic character of the cells [29].

Most recently, a promising method for culturing a self-maintained chicken intestinal epithelial cell line from 19-day-old chicken embryos was published [106]. The cells could survive for 12 days in culture. This is a clear advance in comparison to previous studies, but it should be noted that this is also a relatively short period of time in terms of running multiple series of experiments. For comparison, the IPEC-J2 cell line was already cultivated for up to 98 passages in 2015 [16].

Real-Time-PCR characterization demonstrated the presence of genes for epithelial and staminal markers. Immunofluorescence-centered assays further indicated the presence of epithelial cells through detection of ZO-1, OCLN, villin and cytokeratin 18 while vimentin was not detected [106].

As described above, the barrier function of the epithelium, typically determined by TEER, plays an essential role in the maintenance of intestinal health. In order to also enable TEER measurement in intestinal chicken epithelial cells, the polarization ability of the cell line was investigated and TEER was determined [106]. At seven days after seeding, the TEER reached a value of 64 Ω*cm^2^ and remained at this level until day 12. Compared to human cell lines such as the Caco-2 cell line [107] and the porcine IPEC-J2 cell line, which can reach values of 1000–3000 Ω*cm^2^ when cultured with 5% FBS and 400–500 Ω*cm^2^ when cultured with 10% PS [16], a value of 64 Ω*cm^2^ seems very low. In ex vivo experiments using the Ussing chamber, the TEER of chicken small intestinal epithelium ranged from 149 Ω*cm^2^ [108] to 268 Ω*cm^2^ [109] and that of humans was around 50–100 Ω*cm^2^ [107], suggesting a similar level in vivo.

The low value of 64 Ω*cm^2^ would probably further be more susceptible to fluctuations in the measurement and, in particular, decreases in TEER are more difficult to determine than in cell lines with significantly higher TEER values. However, measurement of TEER was achieved in an intestinal chicken cell line for the first time.

### 3.2. Effect of PFA

In the absence of alternatives, the chicken cell model of Rath and collaborators was used to demonstrate an inhibiting effect of carvacrol on *Campylobacter jejuni* adhesion [98,110]. In primary chicken epithelial cells isolated from small intestine and also only grown for five passages a water-soluble chestnut tannin extract showed to promote proliferation and antioxidant activity while not affecting cellular metabolic rate [104].

## 4. Co-Cultivation and Organoids

The porcine epithelium in vivo arises from epithelial cells and from other cell types such as goblet cells and stem cells [40]. Thus, a clear disadvantage of purely epithelial cell lines such as the IPEC-J2 cell line is their lack of cellular diversity.

To better understand the interplay of different cell types, the co-cultivation of IPEC-J2 cells with other cell types offers a great opportunity. Recently, for example, the direct and indirect co-cultivation of IPEC-J2 cells with monocyte-derived dendritic cells has been studied. The cytokine thymic stromal lymphopoietin (TLSP) is a major component of communication in cultivation without direct cell-to-cell contact [111]. In direct co-cultivation, on the other hand, cell-to-cell contact instead of TLSP has a significant influence on the anti-inflammatory behavior of both cell lines in the unstimulated as well as in the ETEC-challenged state [112]. These results show that the cross-linking of the cells has a great influence on their behavior, which is why the co-cultivation of IPEC-J2 cells with other cell lines of the intestinal epithelium may also provide important new insights for the investigation of the influence of PFA in future.

To enable studies concerning the influence of PFA on the interaction between several different cells and thus to increase the transferability to the situation in vivo, the development of organoids has been strongly promoted in recent years. Intestinal organoids are 3-dimensional cultures of intestinal epithelial stem cells that form a polarized monolayer consisting of the different cell types found in vivo. In addition to their high functional differentiation, their typical architecture with crypts is very similar to the in vivo situation. Organoids provide far-reaching new insights, particularly in research into pathogen–host interaction, and they are also an attractive in vitro models for screening bioactive substances, for example of phytogenic origin. The extensive findings concerning the intestinal organoids of farm animals have recently been well-reviewed [113,114].

## 5. Conclusions

Due to the greater transferability to the human research field, the development of porcine intestinal cell models has been significantly more promoted than those derived from chickens. Moreover, the IPEC-J2 cell line shows the greatest morphological and functional similarity to porcine enterocytes in vivo, and therefore it is predominantly used for the investigation of PFA.

Antimicrobial, anti-inflammatory, anti-oxidative, proliferative and/or barrier function supporting effects have been demonstrated in IPEC-J2 cells for several plant-based feed additives in recent years. It should be critically noted that although the range of substances investigated is high, allowing a comprehensive overview, only few of them have been assessed in more than one study, which limits their reproducibility and applications. Moreover, in the case of PFA obtained directly from fresh plants, the exact extraction procedure is often not specified, which further complicates comparability and reproducibility or even renders it impossible. To verify the results, further in vitro as well as in vivo studies should therefore be conducted with precisely protocolled extracted bioactive substances.

Despite several promising approaches and in particular the first successful measurement of TEER in a chicken cell line, the establishment of a reliable chicken intestinal epithelial cell model faces many challenges and should be the next major step. In particular, a permanent cell line would provide easily accessible and reproducible results in the investigation of PFA.

For both animal types, co-cultivation with other intestinal cells and the use of organoids will provide new insights into the influence of PFA on the interplay of different cell types and tissues in more complex environments.

## Figures and Tables

**Table 1:** Effects of PFA on barrier function of IPEC-J2 cells.

Stimulant/Incubation Time	Substance	Main Results	Reference
DON (0.592 µg/mL, 24 h) POST (2 h)	Resveratrol (22.825 µg/mL)	TEER ↑, paracellular intestinal permeability ↓ Endocytosis and degradation of CLDN-4 and ZO-1 ↓	[58]
-	1. Peppermint oil 2. Spearmint oil (100% pure, 0,25, 50, 100, and 200 µg/mL)	TEER -	[56]
DT2 (0.296 µg/mL DON + 0.002 µg/mL T-2, 48–72 h) POST (24 h)	Rosmarinic acid (18.016 µg/mL)	TEER ↑, IL-6 and IL-8 levels ↓, oxidative stress ↓ CLDN-1 distribution ↑	[59]
-	1. Liquorice (*Glycyrrhiza glabra*) root extract (1000 µg/mL, extracted with water), 2. Angelica (*Angelica archangelica*) root powder (80 µg/mL), 3. Plant powder mix—ground gentian (*Gentiana lutea*) root, ground angelica root, ground cinnamon (*Cinnamomum verum*) bark (80 µg/mL) 4. Oak (*Quercus robur*) bark extract (1000 µg/mL, extracted with ethanol) 5. Milk thistle (*Silybum marianum*) fruit extract, extracted with ethanol (250 µg/mL), ground cinnamon bark (80 µg/mL), ground gentian root (80 µg/mL) All: solubilised in ethanol	1–3: TEER ↑ after 24 h 4: TEER ↓ after 6 h, later TEER - 5: TEER - 1: CLDN-4 ↑	[60]
DON (185 µg/mL) POST (1 h)	Resveratrol (11.413 µg/mL, 12 h)	TEER ↑, translocation of non-pathogenic *E. coli* strain ↓, Paracellular permeability ↓, CLDN-4 ↑	[57]

POST—Treatment post incubation with PFA. Concentrations converted into µg/mL for comparative reasons; ↑: increasing effect; ↓: decreasing effect; -: no effect.

**Table 2:** Effects of PFA on infection of IPEC-J2 cells with intestinal microbial pathogens.

Stimulant/Incubation Time	Substance	Main Results	Reference
1. *Salmonella* Typhimurium (GFP-labelled) 2. *S.* Typhimurium DT104	Prebiotic derived from yellow pine wood (mainly glucose-galactose-mannose-xylose oligomers, steam extraction)	1. *S.* binding (18,000 µg/mL) ↓ 2. *S.* invasion (18,000 or 36,000 µg/mL) ↓	[61]
*S.* Typhimurium ATCC 14,028 (1 h)	1. Cinnamaldehyde (78 µg/mL) 2. Carvacrol (77 µg/mL) 3. Cinnamic acid (1000 µg/mL) All: solubilised in ethanol	1–3.: *S.* invasion ↓ Combination 1, 3, propionic acid: structural impairment fimbriae 1.: HSP70 (immune related gene) ↑ 2.: HSP70 ↑ (only in combination with *S.* Typhimurium and propionic acid)	[62]
*S.* Typhimurium ATCC 14,028 (1 h)	Carvacrol (105, 152–150, 217 µg/mL)	*S*. Adhesion -, *S*. Invasion ↓ Expression porcine β-defensin 2 (pBD-2) ↓	[63]
*E. coli* strain Abbotstown, serotype O147:K89:K88	1. *Lythrum salicaria* herb 2. Its C-glycosylic ellagitannins: Castalagin, vescalagin, salicarinins A and B (extracted from fresh plant)	*E. coli* adhesion ↓ (1. 100 µg/mL, Castalagin 100 µM) TEER ↑ (1. 100 or 500 µg/mL; 2. 20 µM) CLDN 4 (and ZO-1) production ↑ (1. 500 µg/mL)	[64]
*E. coli* K88 (2 h)	Peptides from soybean meal (fermented with *Bacillus subtilis* BS12), alkali extraction method (50 μg/mL)	IL-6, IL-1β, IL-8 ↓ Phosphorylation of NF-κB, IκB-α, and p38 MAPK ↓ TEER ↑, TJ proteins (incl. ZO-1, occludin (OCLN), and CLDN-1) ↑	[65]
*E. coli* strain Abbotstown, serotype O149:K91:F4ac	Additive containing thyme extract (active component thyme EO, 35 g/kg), dilution 10^−2^–10^−5^ (adhesion assay) and 10^−2^–10^−3^ (TEER)	*E. coli* adhesion- TEER-	[68]
*E. coli* ATCC 25922	Epigallocatechin-3-gallate (green tea polyphenol) (22.919 µg/mL), dissolved in DMSO	*E. coli* Translocation ↓; TEER and paracellular permeability - Levels pBD-1 and pBD-2 ↑; pBD-2 ↑ via p38 MAPK pathway	[66]
*E. coli* K88	1. Locust bean (*Ceratonia silique*) exopolysaccharide, wheat bran 2. Soybean hulls, sugar-beet pulp, locust gum, fructooligosaccharides, inulin, mushroom, mannanoligosaccharides, fermented product from *Aspergillus oryzae* All: suspended in phosphate-buffered saline, solid-to-liquid ratio of 1:100, 100 µL on cell culture	1.: *E. coli* adhesion ↓ 2.: *E. coli* adhesion -	[67]

Concentrations converted into µg/mL for comparative reasons; ↑: increasing effect; ↓: decreasing effect; -: no effect.

**Table 3:** Anti-inflammatory effects of PFA on IPEC-J2 cells.

Stimulant/Incubation Time	Substance	Main Results	Reference
1. T2 (0.002 µg/mL) 2. ZEN (3.184 µg/mL) 3. DON (2.963 µg/mL) POST (24 h)	Halophyte extracts (*Convolvulus soldanella*, *Eryngium campestre*, *Galium arenarium*, *Limonium vulgare* and *Ononis spinosa*) (10 µg/mL) extracted from fresh plant (H_2_O:ethanol (1:2))	1–2.: Cell viability ↑ 3.: Cell viability - 1; 3.: TNF-α, IL-8 ↓ 2.: TNF-α, IL-8 -	[76]
LPS (10 µg/mL) POST (2 h)	Eugenol (100 µM (16.42 µg/mL))	IL-8 and TNF-α ↑ TJ proteins (incl. ZO-1 and OCLN), nutrient transporters (B^0^AT1, ASCT2, SGLT1, EAAC1, PepT1) ↑, redistribution ZO-1 ↑	[72]
LPS (0.1 µg/mL) and non- stimulated POST	TCB: Blend of 75% thymol and 25% cinnamaldehyde (0.1 µg/mL)	Permeability ↓, CLDN 4 ↑, Epithelial regeneration ↑, IL-10 ↑; TNF, pIgR ↓	[71]
LPS (10 μg/mL, 12 h) POST (2 h)	Polysaccharides from *Astragalus membranaceus* (200 mg/kg, 2 h)	Phosphorylated p38MAPK, ERK1/2, NF-κB p65 ↓, IκB-α protein ↑	[74]
LPS (5 µg/mL, 1 h) POST (4 h)	Berberine hydrochloride (≥98% purity, dissolved in phosphate buffered saline; 75, 150 and 250 µg/mL)	IL-1β, IL-6 and TNF-α ↓ Key proteins NF- κB/MAPK signalling pathway ↓	[75]
DT2 (0.296 µg/mL DON + 0.002 µg/mL T-2)POST (24 h)	Rosmarinic acid (18.016 µg/mL)	TEER ↑, IL-6 and IL-8 levels ↓, oxidative stress ↓, Distribution CLDN-1 ↑	[59]
LPS (10 μg/mL) POST (1 h)	Thymol (≥98.5%) (7.511 µg/mL)	ROS production ↓, mRNA abundance IL-8 and TNF-α ↓ Expression of nutrient transporters -, TEER ↑ Paracellular permeability ↓, ZO-1 and actin staining ↑	[70]
LPS (10 µg/mL, 6 h), H_2_O_2_ (3.401 µg/mL, 3 h) POST (18 h)	DADS (2.633 µg/mL) and DATS (3.21 µg/mL)	ZO-1 tended to ↑, TEER -, IL-8 ↓	[73]

POST—Treatment post incubation with PFA. Concentrations converted into µg/mL for comparative reasons; ↑: increasing effect; ↓: decreasing effect; -: no effect.

**Table 4:** Anti-oxidative effects of PFA on IPEC-J2 cells.

Stimulant/Incubation Time	Substance	Main Results	Reference
H_2_O_2_ (6.803 µg/mL, 24 h) PRE (24 h)	Pectic polysaccharides from fresh *Codonopsis pilosula* (CPP-1) and *Codonopsis tangshen* (CTP-1) root (20, 10 and 5 μg/mL, 24 h)	Cell viability ↑, T-AOC ↑ Antioxidative enzymes (GSH-Px, SOD, CAT only slightly) ↑ Cellular oxidative damage products (ROS, MDA, LDH) ↓ Transcriptional factor Nrf2 ↑	[85]
H_2_O_2_ (34.015 µg/mL, 1 h) POST (12 h)	*Orthosiphon stamineus* extract (extracted with aqueous ethanol, 50 µg/mL)	TEER ↑, expression ZO-1 ↑, OCLN↑, MDA ↓	[83]
H_2_O_2_ (6.803 µg/mL, 24 h)	Inulin-type fructans from fresh *Codonopsis pilosula* (CPPN) and *Codonopsis tangshen* (CTPN) radix (20, 10 and 5 μg/mL, 24 h)	Cell viability ↑; T-AOC ↑; LDH, MDA ↓; activities GSH-Px, SOD, CAT ↑	[86]
H_2_O_2_ (6.803 µg/mL) POST (12 h)	*Ulva prolifera* extract (mainly polyphenols and unsaturated fatty acids, eluted with 70% methanol from fresh plant; 40 µg/mL)	SOD1, SOD2, CAT ↑; activity GSH-Px1 -Transcriptional factor Nrf2 ↑, ROS ↓, mitochondrial respiration ↑	[84]
1. H_2_O_2_ (17.007 µg/mL, 1 h) 2. DSS (2%, 24 h) POST (3 h)	Combination chestnut (*Castanea sativa* Mill.) tannin extract (50%), quebracho (*Schinopsis* spp.) tannin extract (50%) (dissolved in water, in vitro digested by α-amylase, HCL, pepsin, pancreatin)	Cell viability ↑ (50–400 µg/mL) 1. Oxidative stress ↓ (1200–20 µg/mL) 2. Chemical stress ↓ (50–400 µg/mL)	[82]
-	*Hippophae rhamnoides* polysaccharide from fresh plant (200–600 µg/mL)	ROS, MAD, protein carbonyl ↓; apoptosis ↓ Activities SOD, GSH-Px ↑; CAT relative mRNA levels ↑ IL-1β, IL-2, IL-6, IL-8, TNF-α ↑;	[87]
H_2_O_2_ (500 µM, 4 h) (17.007 µg/mL, 4 h) POST (6 h)	Resveratrol (4.565; 11.413 µg/mL)	Cell viability ↑; levels of CLDN-1, OCLN, ZO-1 ↑ Activities SOD-1, CAT, GSH-Px ↑; apoptosis, ROS↓	[94]
1. Tunicamycin (0.5 μg/mL, 6 h) 2. H_2_O_2_ (17.007 µg/mL, 6 h) PRE	Allicin (2.0 µg/mL)	1. XBP-1s and IRE-1α ↑; GRP78, ATF-4, p-eIF-2α ↓; CHOP -; proliferation -; Apoptosis ↓; ROS ↓; MDA and SOD - Blockage IRE-1α (STF-083010): Reduction GRP78, ATF-4, and p-eIF-2α, ROS ↓ 2. ROS, MDA ↓, SOD ↑	[93]
H_2_O_2_ (17.007 µg/mL) POST (12 h)	Koumine (50, 100 and 200 µg/mL)	Cell viability ↑, apoptosis ↓; ROS, MDA ↓; SOD, CAT, GSH ↑ Mitochondrial membrane potential, Bcl-2 ↑ Activities caspase-9 and -3 ↓	[90]
H_2_O_2_ (12.756–34.015 µg/mL) SIM/POST (2–20 h)	Quercetin (1.25–5 µg/mL)	Cell viability ↑, LDH activity ↓ Apoptosis/necrosis rates changes reversed	[92]
H_2_O_2_ (34.015 µg/mL, 1 h) POST (24 h)	Java tea (*Orthosiphon stamineus*) root extracts (ORE), stem extracts (OSE), and leaf extracts (OLE) (extracted with aqueous ethanol, 50 μg/mL)	Cell viability ↑ ROS ↓	[81]
LPS (10 µg/mL, 6 h), H_2_O_2_ (3.401 µg/mL, 3 h) POST (18 h)	Garlic-derived diallyl disulfide (DADS) and diallyl trisulfide (DATS): (DADS: 2.633 µg/mL/DATS: 3.21 µg/mL)	Catalase activity ↑	[73]
H_2_O_2_ (0.5 or 1 mM, 1 h) (17.007 or 34.0145 µg/mL, 1 h) POST (18 h)	1. Rosmarinic acid (4.504–576.496 µg/mL)2. Quercetin (7.556–241.789 µg/mL)	Cell viability ↑ (1. 50–400 µM; 2. 12.5–200 µM) ROS ↓ (1. 200–600 µM; 2. 25–800 µM) Paracellular permeability ↓, TEER partially ↑	[91]

PRE—Treatment prior to incubation with PFA; POST—Treatment post incubation with PFA, SIM—Simultaneous treatment. Concentrations converted into µg/mL for comparative reasons; ↑: increasing effect; ↓: decreasing effect; -: no effect.

**Table 5:** Impact of PFA on proliferation of IPEC-J2 cells.

Stimulant/Incubation Time	Substance	Main Results	Reference
-	*Perilla frutascens* extracts (0.4 mg/400 µL medium): 1. Extracted with water 2. Extracted with 50% ethanol 3. Extracted with 100% EtOH 4. Extracted with 90% methanol (0.5% formic acid)	Cell proliferation ↓ 67 kDa laminin receptor and cyclin D1 expression (cancer related genes) ↓	[95]
H_2_O_2_ (4 h) (Cell Cycle) POST (3 h)	Quercetin (1.25–5 µg/mL)	G0/G1 phase ↓, S Phase ↑, proliferation index ↑, P 21 and P 27 ↓ Epithelial regeneration ↓	[92]

POST—Treatment post incubation with PFA. Concentrations converted into µg/mL for comparative reasons; ↑: increasing effect; ↓: decreasing effect; -: no effect.

## Data Availability

Not applicable.

## Abbreviations

AMPK: AMP-activated protein kinase; ASCT2: Alanine/serine/cysteine-preferring transporter 2; ATF4: Activating transcription factor 4; B^0^AT1: Sodium-dependent neutral amino acid transporter B^0^AT1; Bcl: B-cell lymphoma; CAT: Chloramphenicol acetyltransferase; CHOP: C/EBP homologous protein; CLDN: Claudin; CPP-1: Pectic polysaccharides from *Codonopsis pilosula* root; CTP-1: Pectic polysaccharides from *Codonopsis tangshen* root; CPPN: Inulin-type fructans from *Codonopsis pilosula* radix; CTPN: Inulin-type fructans from *Codonopsis tangshen* radix; DADS: Garlic-derived diallyl disulfide; DATS: Garlic-derived diallyl trisulfide; DMSO: Dimethyl sulfoxide; DON: Deoxynivalenol; DSS: Dextran sulphide sodium; EAAC1: Excitatory amino acid transporter 3; EHEC: Enterohemorrhagic *E. coli*; ER: Endoplasmatic reticulum; ETEC: enterotoxigenic *E. coli*; FBS: Fetal bovine serum; GLUT2: Glucose transporter 2; GRP78: 78-kDa glucose-regulated protein; GSH-Px: Glutathione peroxidase; HRP: Unsaturated fatty acids and polysaccharides from *Hippophae rhamnoides*; IκB-α: Inhibitor kappa B-alpha; IL: Interleukin; IRE-1α: Inositol-requiring enzyme 1 alpha; JNK: C-Jun-N-terminal kinase; LDH: Lactate dehydrogenase; LPS: Lipopolysaccharide; MDA: Malondialdehyde; MAPK: Mitogen-activated protein kinase; MIP: Macrophage inflammatory protein; mRNA: Messenger ribonucleic acid; MTT: 3-[4, 5-dimethylthiazole-2-yl]-2, 5-diphenyltetrazolium bromide; MUC: Mucin; MYD88: Myeloid differentiation marker 88; Na+/K+-ATPase: Sodium–potassium adenosine triphosphatase; NF-kB: Nuclear factor kappa B; NHE3: Na+/H+ exchanger 3; Nrf2: Nuclear factor erythroid 2-related factor 2; ORC: Oxygen consumption rate; pBD: Porcine beta-defensin; PEDV: Porcine epidemic diarrhea virus; p-eIF-2α: Phosphorylated eukaryotic initiation factor-2 alpha; PepT1: Peptide transporter 1; PFA: Phytogenic feed additives; pIgR: Polymeric immunoglobulin receptor; PS: Porcine serum; ROS: reactive oxidative species; RT-PCR: Reverse transcription-polymerase chain reaction; SGLT: Sodium/glucose cotransporter; SOD: Superoxide dismutase; STEC: Shiga toxin 2e-producing *E. coli*; T-AOC: Total antioxidant capacity; T2: Fusariotoxin T2; TCB: Blend composed of thymol (75%) and cinnamaldehyde (25%); TEER: Transepithelial electrical resistance; TJ: Tight junction; TNF-α: Tumor necrosis factor alpha; TLSP: Thymic stromal lymphopoietin; UPR: Unfolded protein response; X/XO: Xanthine/xanthine oxidase; XBP-1: X-box binding protein 1; ZEN: Zearalenon; ZO-1: Zonula occludens 1.
